# CINOEDV: a co-information based method for detecting and visualizing *n*-order epistatic interactions

**DOI:** 10.1186/s12859-016-1076-8

**Published:** 2016-05-17

**Authors:** Junliang Shang, Yingxia Sun, Jin-Xing Liu, Junfeng Xia, Junying Zhang, Chun-Hou Zheng

**Affiliations:** School of Information Science and Engineering, Qufu Normal University, Rizhao, 276826 China; Institute of Network Computing, Qufu Normal University, Rizhao, 276826 China; Bio-Computing Research Center, Shenzhen Graduate School, Harbin Institute of Technology, Shenzhen, 518055 China; Institute of Health Sciences, Anhui University, Hefei, Anhui 230601 China; School of Computer Science and Technology, Xidian University, Xi’an, 710071 China; College of Electrical Engineering and Automation, Anhui University, Hefei, Anhui 230039 China

**Keywords:** Epistatic interactions, Co-information, Single nucleotide polymorphisms, Particle swarm optimization, Hypergraph

## Abstract

**Background:**

Detecting and visualizing nonlinear interaction effects of single nucleotide polymorphisms (SNPs) or epistatic interactions are important topics in bioinformatics since they play an important role in unraveling the mystery of “missing heritability”. However, related studies are almost limited to pairwise epistatic interactions due to their methodological and computational challenges.

**Results:**

We develop CINOEDV (Co-Information based *N*-Order Epistasis Detector and Visualizer) for the detection and visualization of epistatic interactions of their orders from 1 to *n* (*n* ≥ 2). CINOEDV is composed of two stages, namely, detecting stage and visualizing stage. In detecting stage, co-information based measures are employed to quantify association effects of *n*-order SNP combinations to the phenotype, and two types of search strategies are introduced to identify *n*-order epistatic interactions: an exhaustive search and a particle swarm optimization based search. In visualizing stage, all detected *n*-order epistatic interactions are used to construct a hypergraph, where a real vertex represents the main effect of a SNP and a virtual vertex denotes the interaction effect of an *n*-order epistatic interaction. By deeply analyzing the constructed hypergraph, some hidden clues for better understanding the underlying genetic architecture of complex diseases could be revealed.

**Conclusions:**

Experiments of CINOEDV and its comparison with existing state-of-the-art methods are performed on both simulation data sets and a real data set of age-related macular degeneration. Results demonstrate that CINOEDV is promising in detecting and visualizing *n*-order epistatic interactions. CINOEDV is implemented in R and is freely available from R CRAN: http://cran.r-project.org and https://sourceforge.net/projects/cinoedv/files/.

**Electronic supplementary material:**

The online version of this article (doi:10.1186/s12859-016-1076-8) contains supplementary material, which is available to authorized users.

## Background

Following the development of high-throughput sequencing and genotyping technologies, there has been a rapid increase in the availability of single nucleotide polymorphisms (SNPs). Hence genome-wide association studies (GWAS) have become a routine tool in investigating the genetic architectures of complex diseases, such as cancer, heart disease, diabetes and many others. With these studies, hundreds of thousands of SNPs speculated to associate with complex diseases have been identified. However, these SNPs have been shown to explain only a small proportion of underlying genetic variance of complex diseases, leaving the question of “missing heritability” open for further investigation [1, 2].

Some plausible explanations should be taken into account to reveal the gaps between expectations and realities of GWAS. Firstly, GWAS require p-values (or other similar measures) of disease-associated SNPs to reach a genome-wide significance level after several stringent multiple testing corrections, for example, Bonferroni correction, which may exclude many genuinely associated SNPs that have moderate or weak association signals [3]. Secondly, rare SNPs (i.e., minor allele frequency of each is < 5 %) are difficult to be detected, and sometimes even be ignored in GWAS, though they may play an important role in explaining “missing heritability” [1, 4, 5]. Thirdly, besides SNPs, other types of biological data, for instance, copy number variation, DNA methylation, and gene expression, also provide different, partly independent and complementary, views for unraveling the mystery of “missing heritability” [6, 7]. Fourthly, it is widely believed that nonlinear interaction effects of multiple SNPs or epistatic interactions could unveil a large portion of unexplained heritability of complex diseases [8–11]. In fact, detection of epistatic interactions has already been a compelling step in GWAS [12].

In general, detection of epistatic interactions is of great challenge. The first challenge is the intensive computational burden mainly imposed by the “curse of dimensionality” and the “combinatorial explosion”, which has significant implications for GWAS with millions of SNPs. For instance, search space of a 100 K SNP data set with maximum order of three is an astronomical number ∑_*k* = 1_^3^*C*_100000_^*k*^, where the order refers to the number of SNPs in a SNP combination. The second challenge is the complexity of genetic architecture of a disease. It may involve multiple epistatic interactions interacting with other causative factors in a complicated way, each displaying strong association with the phenotype as a whole but the contained SNPs possibly having small or even no main effects. Limited prior knowledge available for a disease, such as the number of epistatic interactions, the order and the effect magnitude of an epistatic interaction, makes their detection difficult. The third is the association measure that determines how well a SNP combination contributes to the phenotype. A suitable association measure is required to be efficient in computational cost and insensitive to both SNP combination order and effect type, and more importantly, it can truly capture causative epistatic interactions. Though several association measures have been widely used for the detection of epistatic interactions, such as permutation test and *chi-*squared test, developing new association measures that can effectively and efficiently capture epistatic interactions is still a direction. All the above are the great challenges in genome-wide interaction analysis.

Though methodological and computational perplexities of the detection of epistatic interactions have been well recognized, the algorithmic development is still ongoing. Exhaustive methods, e.g., MDR [13], show their successes on small scale data sets. However, for large scale data sets, especially those for GWAS, the detection of epistatic interactions becomes a *needles-in-a-haystack* problem [14] and exhaustive methods are no longer feasible. Recently, heuristic methods are gaining increasing favor since they can retain as many informative SNPs as possible while largely reducing computational complexity. For instance, Zhang et al. developed TEAM [15] to identify epistatic interactions, which updates contingency tables by utilizing a minimum spanning tree. Wan et al. presented an epistatic interaction detection method BOOST [16], which involves only Boolean values and allows the use of fast logic operations to obtain contingency tables. They also proposed another method SNPRuler [17] based on predictive rule inference. Wang et al. used AntEpiSeeker [18] to identify epistatic interactions, which is a two-stage ant colony optimization algorithm. Zhang and Liu developed a Bayesian partition approach BEAM to find groups of genotypes with large posterior probability [19]. Tang et al. introduced the concept of epistatic module and designed a Gibbs sampling approach *epi*MODE [20] to detect such modules, which is a generalization of BEAM.

Besides these methods, co-information based methods appear promising in detecting epistatic interactions since they have a well-developed theory, and can measure multivariate dependence without any complex modeling. Chanda et al. [21] developed a co-information based metric called the interaction index for prioritizing interacting SNPs. They also proposed another three co-information based methods: AMBIENCE [22] and KWII [23] for detecting epistatic interactions associated with the binary phenotype, CHORUS [24] for identifying associations with quantitative traits. Sucheston et al. [25] demonstrated that co-information based methods are flexible and have excellent power to detect epistatic interactions under a variety of conditions that characterize complex diseases.

Although many methods for detecting epistatic interactions have been performed, most of them were constrained to pairwise epistatic interactions, easily ignoring the broader epistasis landscape [26]. Furthermore, these methods usually output identified epistatic interactions, as well as their significance levels, in flat file formats. Hence, the correct understanding of them is sometimes a challenge for researchers, especially for biologists and non-expert users, who are unfamiliar with the methods. A desirable strategy is to develop an effective visualization tool not only to intuitively visualize the detected interactions but also to discover several hidden patterns [27].

However, there have been few studies focused on their visualization. Moore et al. [28] built an interaction graph to visualize detected epistatic interactions. McKinney et al. [29] constructed a genetic association interaction network (GAIN) to characterize detailed interactions, whose edges quantify the synergy between pair SNPs with respect to the phenotype. GAIN has been successfully used for identifying modulators of antibody response to smallpox vaccine [30], GWAS of bipolar disorder [31], and analyzing exome data for systemic lupus erythematous cases and controls [32]. Hu et al. [33] proposed a statistical epistasis network (SEN) approach, which has been proven to be able to discover pairwise epistatic interactions of bladder cancer [34, 35] and prostate cancer [36]. They also demonstrated that SEN supervised search is able to infer several 3-order epistatic interactions with significantly high associations at a substantially reduced computational cost [37]. Though these network-assisted methods can provide a global map of pairwise epistatic interactions, can indirectly capture higher order epistatic interactions on the basis of observing topology structures of networks, and can be exported for visualization in existing tools, such as Cytoscape and Graphviz, they could not directly detect and visualize *n*-order epistatic interactions, for example, *n* = 3 or larger. More recently, Hu et al. [38] presented a visualization tool ViSEN, which can show both pairwise and 3-order epistatic interactions, in addition to main effects, in one network. To the best of our knowledge, it is the first visualization tool that shows three orders of effects simultaneously. Nevertheless, different orders of effects in ViSEN are difficult to be fairly and intuitively compared. Wu et al. [27] designed another visualization tool EINVis to analyze and explore genetic interactions, which utilizes a tree ring view to simultaneously visualize the hierarchical interactions between SNPs, genes, and chromosomes. However, EINVis is limited in detecting and visualizing high order epistatic interactions.

In the light of above observations, we develop CINOEDV (Co-Information based NOrder Epistasis Detector and Visualizer) for the detection and visualization of epistatic interactions of their orders from 1 to *n* (*n* ≥ 2). CINOEDV is composed of two stages, namely, detecting stage and visualizing stage. In detecting stage, co-information based measures are employed to quantify association effects of *n*-order SNP combinations to the phenotype, and two types of search strategies are introduced to identify *n*-order epistatic interactions: an exhaustive search for lower order epistatic interactions and/or small scale data sets, a particle swarm optimization (PSO) based search for higher order epistatic interactions and/or large scale data sets. In visualizing stage, all detected *n*-order epistatic interactions are used to construct a hypergraph, where a real vertex represents the main effect of a SNP and a virtual vertex denotes the interaction effect of an *n*-order epistatic interaction. By deeply analyzing the constructed hypergraph, some hidden clues for better understanding the underlying genetic architecture of complex diseases could be revealed, for instance, higher order epistatic interactions, hub SNPs and connected subgraphs. Experiments of CINOEDV and its comparison with state-of-the-art methods are performed on lots of simulation data sets under the evaluation measures of both detection power and computational complexity. Results demonstrate that CINOEDV is promising in detecting and visualizing *n*-order epistatic interactions. In addition, CINOEDV is also applied on a real data set of age-related macular degeneration (AMD), and results of which provide several new clues for the exploration of causative factors of AMD. CINOEDV might be an alternative to existing methods for the detection and visualization of *n*-order epistatic interactions.

## Methods

### Co-information based association measures

Before introducing the measures, several terms and notations are described. At present, the generally accepted way of mapping SNPs is to collect them as a matrix, where a row represents genotypes of an individual and a column represents a SNP. Genotypes of a SNP are coded as {0, 1, 2}, corresponding to homozygous common genotype, heterozygous genotype, and homozygous minor genotype. The label of an individual is a binary phenotype being either 0 (control) or 1 (case). Based on this numerical mapping, let *N* and *M* be the number of SNPs and the number of individuals in the data respectively. Below we will discuss the definitions of co-information based association measures between *n* SNPs *S*_1_, ⋯, *S*_*n*_, that randomly sampled from *N* SNPs, and the phenotype *C*.

Co-information is one of several generalizations of mutual information, and can measure multivariate dependence without any complex modeling [39]. Co-information among *n* SNPs and the phenotype *C* is defined as an alternating sum of the joint entropies of all possible subsets *T* of *V* using the difference operator notation of Han [40],

$$ CI\left({S}_1;\cdots; {S}_n;C\right)=-{{\displaystyle \sum_{T\subseteq V}\left(-1\right)}}^{n+1-\left|T\right|}H(T), $$

where *V* = {*S*_1_, ⋯, *S*_*n*_, *C*}, *T* represents all possible subsets of *V*, and *H*(*T*) is the joint entropy of *T*, which can be written as

$$ H(T)=-{\displaystyle \sum_{t\in T}p(t)} \log p(t), $$

and *p*(*t*) is the probability mass function.

It is seen that co-information is a parsimonious, multivariate measure quantifying interactions that cannot be obtained without observing all variables at the same time [24], and it seems promising for detecting *n*-order epistatic interactions. However, it also has two confusing properties retarded its wider adoption as an association measure.

The first is its value. In the bivariate case, co-information is equivalent to mutual information and its value is always positive. But in the multivariate case, its value can be positive or negative, the interpretation of which is generally intuitive [26]: a positive value is an evidence of interactions among variables; a negative value indicates the presence of redundancy; and a value of zero denotes that variables are independent or, more likely, interact with a mixture of synergy and redundancy. Almost all existing applications of co-information depend upon this intuitive explanation [21, 24, 28, 29, 33, 38], and it is also the basis of our association measures.

The second is its sensitivity to the SNP combination order. This property leads to difficulty in ranking SNP combinations of different orders. As yet, it still lacks the widely accepted normalization method. In this study, we make use of the order-fixed averages of co-information values to normalize them, defined as *n*-order interaction effect,

$$ NCI\left({S}_1;\cdots; {S}_n;C\right)=\frac{CI\left({S}_1;\cdots; {S}_n;C\right)}{H(C)}\cdot \frac{\overline{C{I}_1}}{\overline{C{I}_n}}, $$

where *H*(*C*) is the entropy of the phenotype, $\overline{C{I}_n}$ and $\overline{C{I}_1}$ are respective averages of all considered *n*-order and 1-order co-information values. The first part of the formula provides the percentage of explaining the phenotype by giving the knowledge of *n* SNPs, and the second part is a coefficient that balances the contributions of SNP combinations of different orders to the phenotype.

However, *NCI* only measures contribution of a SNP combination itself, not containing contributions of its subsets. In fact, the effect of an *n*-order SNP combination to the phenotype consists of main effects of all involved SNPs, as well as interaction effects of itself and its all subsets. To quantify the total contribution of a SNP combination to the phenotype, another co-information based association measure is presented, defined as the summation of all involved contributions, including its contribution, and contributions of its subsets whose *NCI* values reach the user-specified thresholds. The formula can be written as

$$ CCI\left({S}_1;\cdots; {S}_n;C\right)={\displaystyle \sum_{Z\subseteq CS\cap Z\subseteq \left\{{S}_1;\cdots; {S}_n\right\}}NCI\left({Z}^{\prime };C\right)}, $$

where *Z*^′^ represents all SNPs in the set *Z*, *C* represents the phenotype, and *CS* is a set of SNP combinations that their *NCI* values pass the user-specified thresholds.

### Search strategies

CINOEDV supports two types of search strategies, with *NCI* as its association measure, to simultaneously detect epistatic interactions of their orders from 1 to n (*n* ≥ 2). One is an exhaustive search for lower order epistatic interactions and/or small scale data sets. With genome wide SNPs from thousands of individuals, it is difficult to search high order epistatic interactions exhaustively because of their heavy computational burden. CINOEDV provides a PSO based search for higher order epistatic interactions and/or large scale data sets.

The PSO is a popular member of swarm intelligence algorithms inspired by the collective behaviors of organisms, like birds (viewed as particles), which can jointly perform many complex tasks though each individual is very limited in its capability [41]. In PSO, the position of a particle represents a possible solution which is adjusted according to its velocity, and estimated by a fitness function at each generation. A higher fitness value implies a better position. The velocity of a particle is updated according to three factors: its previous velocity, its individual experience, and the common knowledge of the swarm. The individual experience of a particle is the best position that it has travelled. The common knowledge of the swarm is the best one among individual experiences of all particles. This feedback strategy leads the swarm gradually converge to an optimal solution [42].

In our PSO based search, *NCI* is applied as its fitness function. That is to say, a higher *NCI* value indicates a stronger association between the SNP combination and the phenotype. Compared with the PSO, our PSO based search has its own highlights: detecting multiple epistatic interactions with different orders at the same time, dynamic inertia weight, and opposition based learning.

Suppose $Positio{n}_g(q)=\left({S}_{q1}^g,\cdots, {S}_{qk}^g,\cdots, {S}_{q{K}_q}^g\right)$ is the position of the *q*_*th*_ particle at iteration *g*, where *q* ∊ {1, ⋯, *Q*}, *g* ∊ {1, ⋯, *G*}, *k* ∊ {1, ⋯, *K*_*q*_}, *S*_*qk*_^*g*^ is the selected *k*_*th*_ SNP of the *q*_*th*_ particle at iteration *g*, *Q* is the number of particles, *G* is the number of iterations, and *K*_*q*_ is the considered order of epistatic interactions of the *q*_*th*_ particle, which is randomly specified within [1, *n*] at the initialization stage. The velocity of the *q*_*th*_ particle at iteration *g* is denoted as $Velocit{y}_g(q)=\left({v}_{q1}^g,\cdots, {v}_{qk}^g,\cdots, {v}_{q{K}_q}^g\right)$, where *v*_*qk*_^*g*^ is the velocity of *S*_*qk*_^*g*^. The individual experience of the *q*_*th*_ particle is written as $Pbes{t}_g(q)=\left(P{S}_{q1}^g,\cdots, P{S}_{qk}^g,\cdots, P{S}_{q{K}_q}^g\right)$. The common knowledge of the swarm is redefined as the best ones among individual experiences of particles with the same considered orders, i.e., *Gbest*_*g*_^*K*^ = (*GS*_1_^*g*^, ⋯, *GS*_*k*_^*g*^, ⋯, *GS*_*K*_^*g*^), where *K* ∊ [1, *n*]. During the initialization stage, *Position*_1_(*q*), *Velocity*_1_(*q*), *Pbest*_1_(*q*) and *Gbest*_1_^*K*^ are randomly initialized in their respective domains.

The PSO based search detects epistatic interactions by continuously updating velocity and position of each particle at all iterations. The velocity of *S*_*qk*_^*g*^ is updated according to the following two equations,

$$ {\tilde{v}}_{qk}^{g+1}={W}_{qk}^g\cdot {v}_{qk}^g+{c}_1\cdot {r}_1\cdot \left(P{S}_{qk}^g-{S}_{qk}^g\right)+{c}_2\cdot {r}_2\cdot \left(G{S}_k^g-{S}_{qk}^g\right), $$

$$ {v}_{qk}^{g+1}=\left\{\begin{array}{cc}\hfill {\tilde{v}}_{qk}^{g+1}\hfill & \hfill {\tilde{v}}_{qk}^{g+1}\in \left[1-N,N-1\right]\hfill \\ {}\hfill rand\left(1-N,N-1\right)\hfill & \hfill {\tilde{v}}_{qk}^{g+1}\notin \left[1-N,N-1\right]\hfill \end{array}\right., $$

where acceleration factors *c*_1_ and *c*_2_ control how far a particle moves in a single iteration, *r*_1_ and *r*_2_ are random values in (0, 1), *GS*_*k*_^*g*^ is the *k*_*th*_ SNP of $Gbes{t}_g^{K_q}$ (*K* = *K*_*q*_) at iteration *g*, *W*_*qk*_^*g*^ is the inertia weight regulating the impact of the previous velocity of a particle on its current velocity.

For the inertia weight, a large weight facilitates the global exploration and thus enables the method to execute a search over various regions, while a small weight facilitates the local exploitation, which helps to search a promising region. In order to balance the global exploration and the local exploitation, a dynamical inertia weight is introduced, defined as

$$ {W}_{qk}^g=\frac{ \max \left( coun{t}_g\right)- coun{t}_g\left[P{S}_{qk}^g\right]}{ \max \left( coun{t}_g\right)- \min \left( coun{t}_g\right)}, $$

where *count*_*g*_ = (*ct*_1_^*g*^, ⋯, *ct*_*m*_^*g*^, ⋯, *ct*_*N*_^*g*^), and *ct*_*m*_^*g*^ is a counter that counts the number of SNP *m* presented in *Pbest* from iteration 1 to iteration *g*. This strategy allows particles to cover a wider search space while the considered SNP is likely to be a random one, and to converge on a promising region of the search space while capturing a highly suspected SNP.

Based on *v*_*qk*_^*g*+1^, the position of *S*_*qk*_^*g*^ is updated to *S*_*qk*_^*g*+1^ using the following two equations,

$$ {\tilde{S}}_{qk}^{g+1}={S}_{qk}^g+{v}_{qk}^{g+1}, $$

$$ {S}_{qk}^{g+1}=\left\{\begin{array}{cc}\hfill \operatorname{int}\left({\tilde{S}}_{qk}^{g+1}\right)\hfill & \hfill {\tilde{S}}_{qk}^{g+1}\in \left[1,N\right]\hfill \\ {}\hfill \operatorname{int}\left( rand\left(1,N\right)\right)\hfill & \hfill {\tilde{S}}_{qk}^{g+1}\notin \left[1,N\right]\hfill \end{array}\right., $$

where *rand*( ⋅ ) is the random function and int(⋅) is the rounding function. Random functions used for updating both *v*_*qk*_^*g*+1^ and *S*_*qk*_^*g*+1^ help to increase the diversity of the search.

Another highlight introduced to the PSO based search is the opposition based learning, basic principle of which is the consideration of a solution and its corresponding opposite solution simultaneously to approximate the global optima [43]. In our PSO based search, if the solution is *Position*_*g*_(*q*), its corresponding opposite solution is defined as

$$ Positio{n}_g^{\hbox{'}}(q)=1+N- Positio{n}_g(q), $$

which not only expands the search space and enhances the global explorative ability, but also accelerates the convergence and avoids premature convergence.

By comparing *NCI* values of *Position*_*g*_^’^(*q*), *Position*_*g*_(*q*) and *Pbest*_*g*_(*q*), the individual experience of the *q*_*th*_ particle at iteration *g* + 1, i.e., *Pbest*_*g*+1_(*q*), is updated to the best one among them. Similarly, whether the common knowledge of the swarm at iteration *g* + 1, e.g., *Gbest*_*g*+1_^*K*^, is updated or maintained as *Gbest*_*g*_^*K*^ depends on individual experiences of particles with the same order *K*. Specifically, *Gbest*_*g*+1_^*K*^ is updated to *Pbest*_*g*+1_(*q*) while *NCI* value of *Pbest*_*g*+1_(*q*) is the highest one among those of individual experiences of particles with the order *K*, and is also higher than that of *Gbest*_*g*_^*K*^. When completing the iteration process, the PSO based search reports the sorted *Pbest*_*G*_ according to their descending *NCI* values as its detected epistatic interactions.

### Epistasis hypergraph and deep analyses

In visualizing stage, lists of all epistatic interactions identified in detecting stage, or similar lists generated by other methods, are exported to construct an undirected epistasis hypergraph for refining the interpretation of genetic basis of disease susceptibility and disease etiology, by capturing and visualizing broader epistasis landscape. The hypergraph is composed of weighted vertices and unweighted edges. For weighted vertices, two types of them are presented, that is, real vertices and virtual vertices. A real vertex represents a SNP and its weight is the *NCI* value between the SNP and the phenotype, corresponding to the main effect of the SNP to the phenotype. A virtual vertex denotes the *n*-order interaction effect of the combination of linking SNPs to the phenotype, and its weight is the *NCI* value between the SNP combination and the phenotype. In the hypergraph, each red circle is a real vertex in which SNP name or index is labeled, each non-red circle is a virtual vertex. Sizes of vertices are respectively in proportion to their weights. For unweighted edges, each of them links a SNP and an effect of this SNP to the phenotype. From the hypergraph, effects of different orders, especially high orders, as well as topology structures of SNPs, can be intuitively visualized and compared.

For deep analyses of the constructed hypergraph, several useful tools are also provided in CINOEDV. For example, epistatic interactions can be visually displayed according to their descending effects, either *NCI* values or *CCI* values; penetrance of an epistatic interaction can be estimated and visualized; degree of a real vertex in the hypergraph and connectivity of the hypergraph can be further analyzed. More details about these tools are available in its user manual. With the help of these tools, some hidden clues for better understanding the underlying genetic architecture of complex diseases could be revealed.

## Results and Discussion

### Experiments on simulation data

#### Detection power analysis for pairwise epistatic interactions

**Fig. 1:**
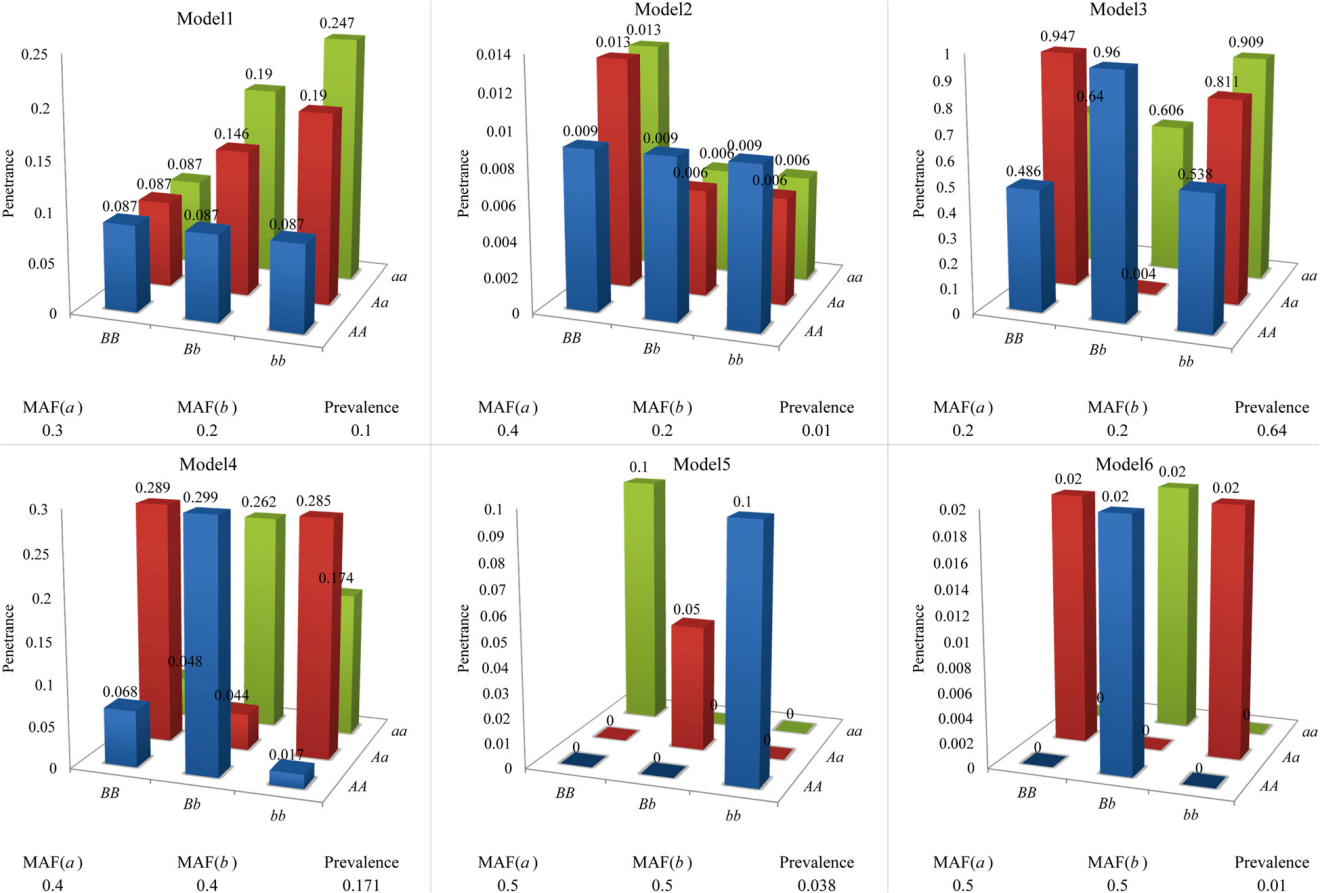
Six models of epistatic interactions. Model1 and Model2 are models displaying both marginal effects and interaction effects, and Model3 ~ Model6 show no marginal effects but interaction effects. Specifically, the penetrance in Model1 increases only when both SNPs have at least one minor allele [19, 20]; Model2 assumes that the minor allele in one SNP has the marginal effect, however, the effect is inversed while minor alleles in both SNPs are present [19]; Model3 and Model4 are directly cited from the reference [55]; Model5 is a ZZ model [56]; and Model6 is an XOR model [55]. Penetrance is the probability of the occurrence of a disease given a particular genotype. Prevalence is the proportion of individuals that have a disease. MAF(*a*) and MAF(*b*) are minor allele frequencies of *a* and *b*

Six commonly used models of epistatic interactions are simulated for the study. Details of these models are given in Fig. 1. For each model, 200 data sets are generated by the simulator *epi*SIM [44], which describes the simulation steps of SNP data sets as well as true epistatic interactions of SNPs in detail, and has been used in several references [26, 45, 46]. Among them, each data set contains 2000 cases and 2000 controls. In the first 100 data sets, 100 SNPs are genotyped, while in other 100 data sets, the number of SNPs is increased to 10000, which simulates high dimensional data sets like those in GWAS. In addition, three types of detection power are introduced to evaluate the performance of CINOEDV, definitions of which are given in our previous studies [26, 47, 48] and the Additional file 1: Note 1.

Detection power of CINOEDV is evaluated by comparative studies with several existing state-of-the-art methods, using above simulation data. They are TEAM, BOOST, SNPRuler, AntEpiSeeker, and *epi*MODE. These methods are recently proposed, claimed to facilitate the detection of epistatic interactions. Their packages and manuals are available online [47], where default parameter settings, as well as parameter adjustment strategies, are described in detail. In the study, parameters of these methods are generally set as default. Only a few are modified according to suggestions in their respective manuals in order to ensure a fair comparison. For TEAM, permutation number is set to 100. For BOOST, interaction threshold is set to 10, i.e., results of BOOST are the epistatic interactions whose likelihood ratio test statistic values >10 with 4 degrees of freedom. For AntEpiSeeker, the numbers of ants and iterations are set to 500 and 10, respectively. For *epi*MODE, iteration number is set to 100. For CINOEDV, both the exhaustive search (CINOEDV(E)) and the PSO based search (CINOEDV(P)) are evaluated, and top 10 identified epistatic interactions are recorded for each run. For CINOEDV(P), the number of particles is set to 500, and the number of iterations is set to 10, which are the same as those of AntEpiSeeker for a fair comparison.

**Fig. 2:**
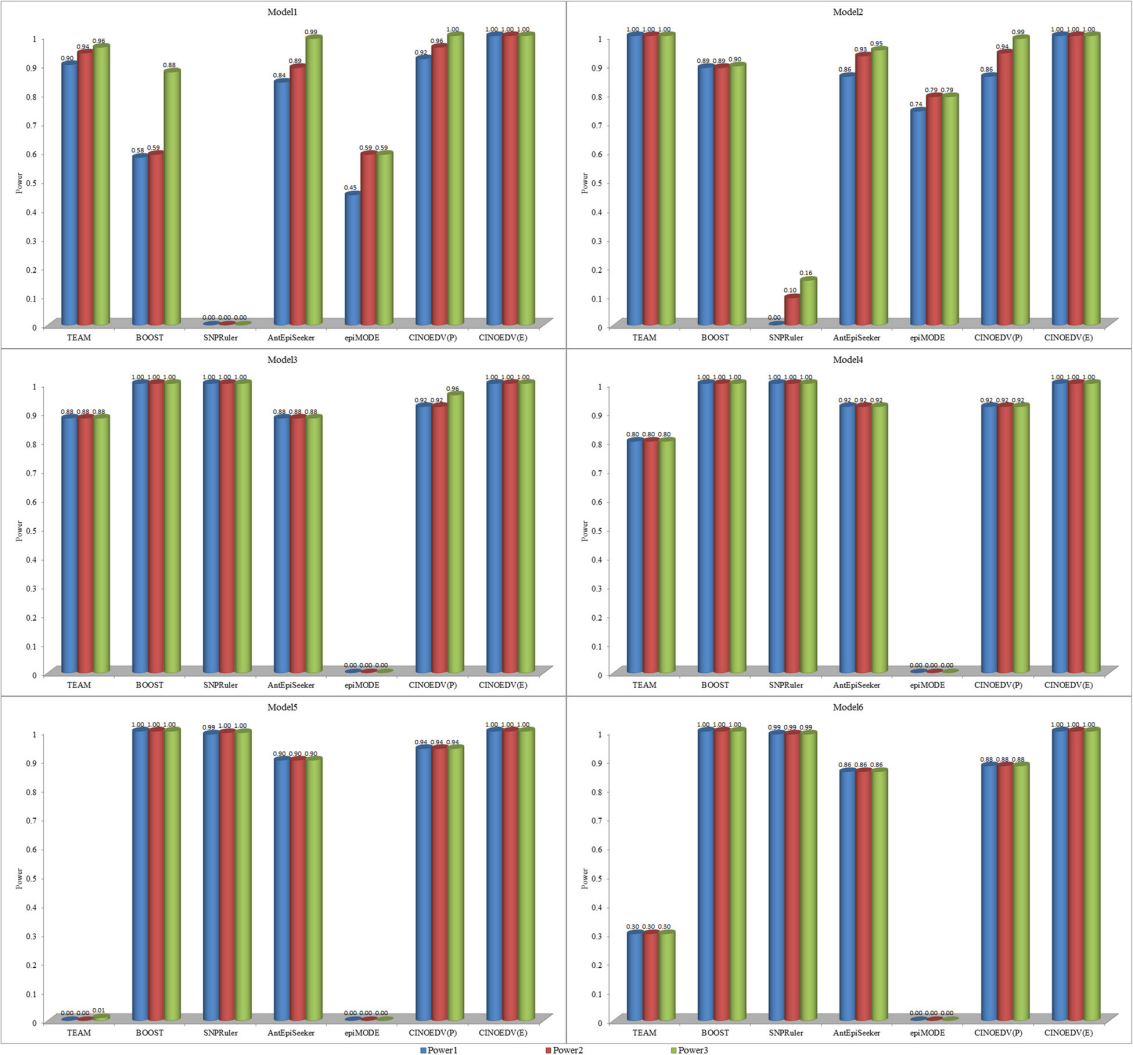
Detection power of compared methods on 100-SNP data sets

**Fig. 3:**
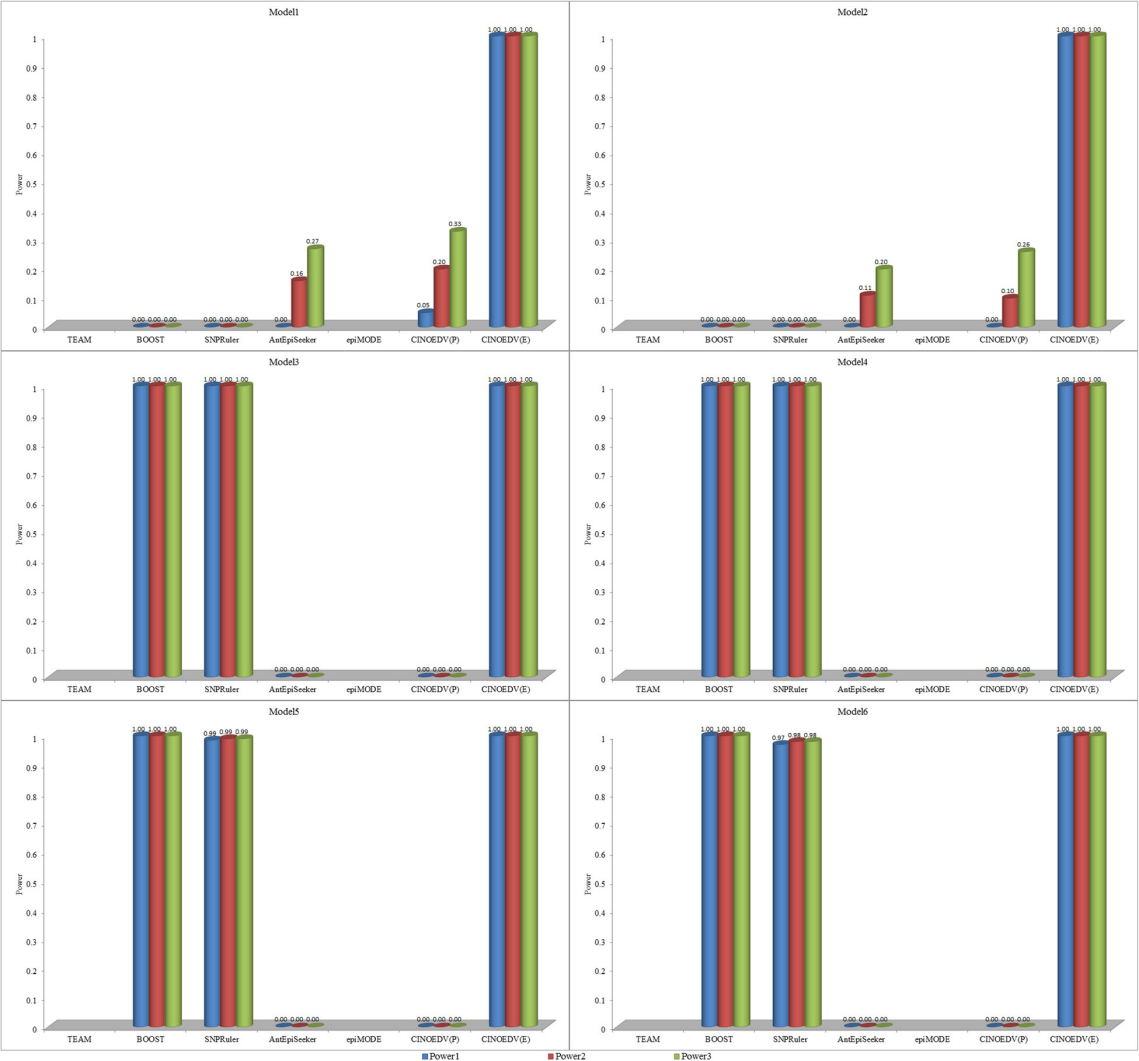
Detection power of compared methods on 10000-SNP data sets. TEAM and *epi*MODE are not considered here due to their unaffordable computational cost on high dimensional data sets

Detection power of compared methods on 100-SNP data sets is shown in Fig. 2, and that on 10000-SNP data sets is shown in Fig. 3. It is seen that CINOEDV is promising in detecting epistatic interactions. Specifically, CINOEDV(E) identifies all epistatic interactions and outperforms other methods on all cases regardless of models and SNP sizes; detection power of CINOEDV(P) on almost all models of 100-SNP data sets is comparable and sometimes superior to that of compared methods; among models of 10000-SNP data sets, though CINOEDV(P) has moderate detection power on Model1 and Model2, and detects nothing on other models, it is still the runner-up; the decrease of detection power of CINOEDV(P) from 100-SNP to 10000-SNP data sets is because of the inevitably increased search space and the non-change parameter settings; compared with detection power of other methods on different models, detection power of CINOEDV on different models is much more stable, implying that CINOEDV is not sensitive to model types; for Model1 and Model2, Power1, Power2 and Power3 of a compared method usually have different values since these models displaying not only interaction effects but also marginal effects, leading to the method sometimes only identifying several ground-truth SNPs but not epistatic interactions, where ground-truth SNPs refer to the SNPs in models; similarly, for each method on Model3 ~ Model6, Power1, Power2 and Power3 are almost equal because single ground-truth SNPs show no main effects.

For compared methods, their results are consistent with and complementary to previous reported results [47, 48]. In terms of detection power analysis for pairwise epistatic interactions, BOOST performs best in most cases, especially on Model3 ~ Model6 since it is a model-based method that only focuses on identifying models displaying no marginal effects but interaction effects like Model3 ~ Model6. However, BOOST is constrained to pairwise epistatic interactions, can not infer high order epistatic interactions and graph-structure interactions, is incapable of visualizing epistatic interactions with different orders in the hypergraph, which are just highlights of CINOEDV that will be discussed later.

#### Inferring higher order epistatic interactions from hypergraph

For assessing the capability of CINOEDV in inferring higher order epistatic interactions from the epistasis hypergraph, four models are used that have been developed previously [49, 50], namely, *Three* − 1, *Three* − 2, *Four* and *Five. Three* − 1 is a model of 3-order epistatic interaction displaying both marginal effects and interaction effects. *Three* − 2 is a pure model of 3-order epistatic interaction, where the association to the phenotype is only observable when all 3 ground-truth SNPs are considered together, that is, no main effects and no pairwise epistatic interactions. Similarly, *Four* and *Five* are models of 4-order and 5-order epistatic interactions, each displaying no main effects and no 2-order interaction effects. For each corresponding data set also generated by *epi*SIM [44], 1500 cases and 1500 controls are included and genotyped by 1000 SNPs.

**Fig. 4:**
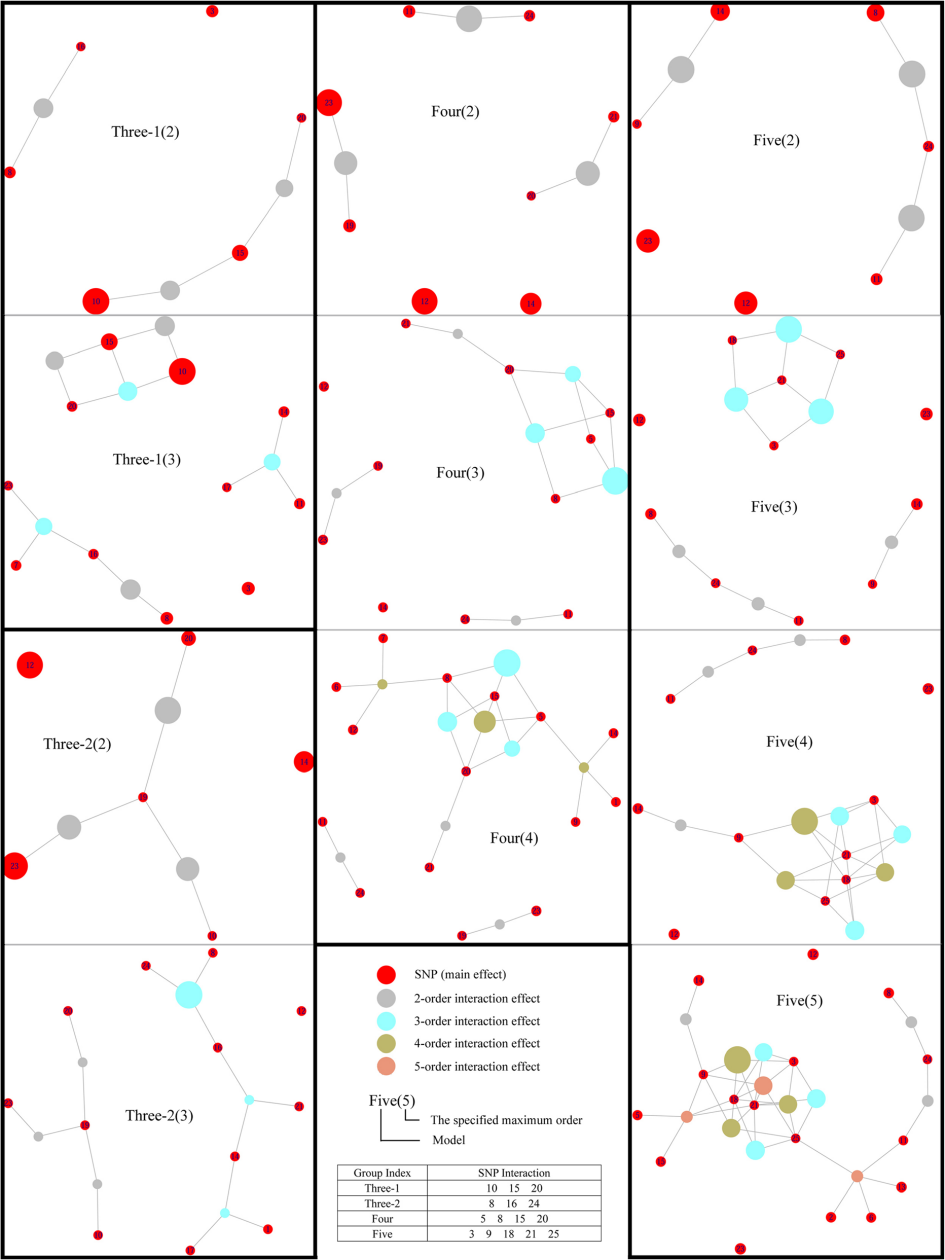
Hypergraphs of compared data sets. Indices of ground-truth SNPs of each model (Group Index) are recorded in the table (SNP Interaction). *Three-1* and *Three-2* are models of 3-order epistatic interaction: the former displaying both marginal effects and interaction effects, and the latter being a pure model. Sizes of vertices are respectively in proportion to their effects to the phenotype

We apply CINOEDV(E) on these data sets with the specified maximum order from 2 to *n*, where *n* is the order of embedded epistatic interaction. Their hypergraphs are shown in Fig. 4, from which, we have the following observations.

Though *Three* − 1 is a model of 3-order epistatic interaction, it is able to be inferred from the epistasis hypergraph *Three* − 1(2), which is constructed only by main effects and 2-order interaction effects. In the *Three* − 1(2), two strong 2-order interaction effects linking 3 SNPs, 2 of which show much stronger main effects than others, forms a connected subgraph. Under the hypothesis that the sets of SNPs that are linked together by strong low order interaction effects in the hypergraph may indicate the existence of higher order epistatic interactions, we could infer that these 3 SNPs in the connected subgraph might jointly modify the phenotype. In reality, they are indeed ground-truth SNPs of the model *Three* − 1, demonstrating that the inference is correct. In the *Three* − 1(3), the topology structure of the 3-order epistatic interaction becomes clear: besides 2 strong main effects and 2 strong 2-order interaction effects, they also display a strong 3-order interaction effect. For models *Three* − 2, *Four* and *Five*, they are difficult to be inferred from their respective hypergraphs *Three* − 2(2), *Four*(2), and *Five*(2) because there are no main effects and no pairwise epistatic interactions in these models. In the *Three* − 2(3), the combination of 3 ground-truth SNPs only shows a 3-order interaction effect, and this effect is far stronger than other effects, demonstrating that *Three* − 2 is a pure model of 3-order epistatic interaction. Similarly, using the same inference strategies, we could infer *Four* and *Five* from their hypergraphs *Four*(3) and *Five*(4) perfectly, which implies that epistasis hypergraph constructed by main effects and low order interaction effects is a promising guide map for capturing higher order epistatic interactions while substantially reducing computational cost. In addition, 4 out of 5 ground-truth SNPs could be inferred in the *Five*(3) since they make up of an attractive connected subgraph, having 3 strong 3-order interaction effects. From these hypergraphs, we can conclude that an epistatic interaction is usually characterized as a connected subgraph or part of a connected subgraph, where vertices interact with each other more closely. These observations show that CINOEDV is capable of inferring higher order epistatic interactions from the epistasis hypergraph.

Besides, four state-of-the-art network-assisted methods, that is, GAIN, SEN, ViSEN, and EINVis, are also applied on these data sets for the comparative analyses, results of which are recorded in Additional file 1: Figure S1-S4. It is seen that GAIN, SEN, and EINVis focus on the visualization of pairwise epistatic interactions and only *Three* − 1 can be inferred due to its strong marginal effects. ViSEN is able to show three orders of effects at the same time, and can detect models of *Three* − 1, *Three* − 2, and *Four* perfectly, as well as 4 ground-truth SNPs out of 5 in model *Five*. Nevertheless, different orders of effects in ViSEN are difficult to be fairly and intuitively compared.

#### Computational complexity analysis

The main purpose of CINOEDV is to identify multiple epistatic interactions with different orders from genome wide data. Just because of this, computational efficiency is a key issue that has to be considered. We use 100-SNP and 10000-SNP data sets that have been simulated before to compare computational efficiency with TEAM [15], BOOST [16], SNPRuler [17], AntEpiSeeker [18], *epi*MODE [20], CINOEDV(P) and CINOEDV(E). For a fair comparison, parameters of them are set as those values discussed previously. Experiments are conducted with Intel Xeon 2.00 GHz CPUs and 6 GB of RAM running Microsoft Windows XP Professional x64 Edition 2003 Service Pack 2. The average running time of compared methods on these data sets is recorded in Table 1.

**Table 1:** Average running time (seconds) of compared methods on simulation data sets. The method *epi*MODE could not deal with data sets with 10000 SNPs at affordable time cost

Methods	TEAM	BOOST	SNPRuler	AntEpiSeeker	*epi*MODE	CINOEDV(P)	CINOEDV(E)
100-SNP data sets	13.14	0.36	1.56	1146.60	50.46	48.05	23.94
10000-SNP data sets	41742.00	248.52	3495.60	6252.00	>41742.00	76.38	4872.50

For CINOEDV(P), its average running time on 100-SNP data sets is slow, just faster than that of AntEpiSeeker and *epi*MODE, even slower than that of CINOEDV(E). This is because its search space, controlled by the numbers of particles and iterations, is very close to the space of exhaustive search, and it also needs extra time to deal with the particle updating process. On 10000-SNP data sets, CINOEDV(P) is the fastest one among compared methods since its search space is not changed. CINOEDV(P) can finish the search at affordable time cost, and this time cost can be estimated and controlled by setting its parameters freely, which enable it facilitate searching epistatic interactions in large scale data sets. For CINOEDV(E), no matter on 100-SNP or 10000-SNP data sets, its detection power is perfect, however, the exhaustive search is heavy in time cost.

The bright spot of CINOEDV in computation complexity is that the hypergraph constructed by main effects and low order interaction effects is able to supervise the search for higher order epistatic interactions at a substantially reduced computational cost. Even if the computational complexity of building a hypergraph is considered together, the computational cost is still far less than that of the exhaustive search. This reduction of computational complexity is even more encouraging in the era of GWAS.

### Application to AMD data

CINOEDV, as well as other competing methods, including SNPRuler, AntEpiSeeker, BEAM, *epi*MODE, and BOOST, are also applied on a real AMD data set [51], which contains 103611 SNPs genotyped with 96 cases and 50 controls. AMD is the most important cause of irreversible visual loss in elderly populations, and has been considered as a genetic disease where multiple epistatic interactions are exist [20, 26].

**Fig. 5:**
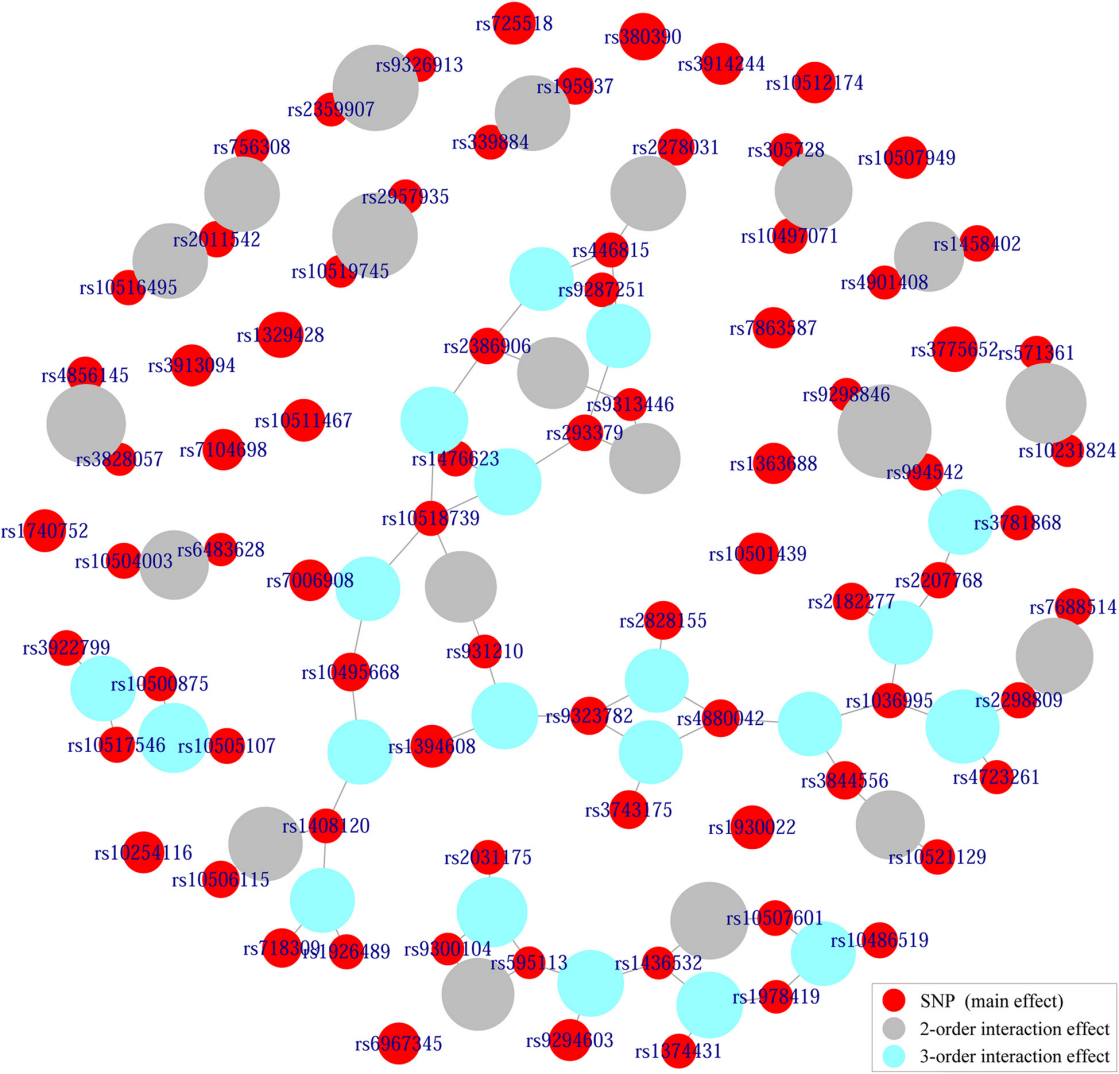
Epistasis hypergraph of AMD data set. SNP names are labeled in their corresponding vertices. Sizes of vertices are respectively in proportion to their effects to the phenotype

We use the PSO based search to explore epistatic interactions with the maximum order specified to 3, where the numbers of particles and iterations are set to 50000 and 1000. Top 20 SNPs with high main effects, top 20 epistatic interactions with high 2-order interaction effects, and top 20 epistatic interactions with high 3-order interaction effects are reported in Additional file 1: Table S1-S3, respectively. The epistasis hypergraph built by these effects is shown in Fig. 5. Besides, detected SNPs and epistatic interactions of other competing methods are recorded in Additional file 1: Table 4.

It has been widely accepted that rs380390 and rs1329428 are believed to be significantly associated with AMD [20]. These two SNPs are in an intron of the *CFH* gene. *CFH* is a regulator that activates the alternative pathway of the complement cascade, the mutations in which can lead to an imbalance in normal homeostasis of the complement system. This phenomenon is thought to account for substantial tissue damage in AMD. rs1394608, that has been implicated in AMD [20], resides the intron of *SGCD* gene, variants of which regulate the degradation of extracellular matrix by facilitating access of other degradative matrix enzymes, thus resulting in the pathological extracellular deposits in retinal. Our method, BOOST and *epi*MODE confirm these three SNPs successfully. BEAM identified both rs380390 and rs1329428, but did not detect rs1394608. AntEpiSeeker only found rs380390, and SNPRuler did not identify these three SNPs at all.

The SNP combination rs994542:rs9298846, that identified by both CINOEDV and BOOST, has the strongest 2-order interaction effect, though each of them shows small main effect, implying that they might be a pure epistatic interaction [52]. Most SNPs in Additional file 1: Table S1 have been reported in previous AMD association studies [20, 53, 54]. But on the contrary, almost no SNPs in Additional file 1: Table S2 and S3 have been identified previously. This might be because existing AMD related GWAS mainly focus more on identifying SNPs with strong main effects, and SNPs in Additional file 1: Table S2 and S3 are weak in main effects, showing strong interaction effects through their combinations. These SNPs and their combinations need further studies with the use of large scale case–control samples to confirm whether they have true associations with AMD.

In Fig. 5, SNPs are grouped into maximal connected subgraphs, which may indicate that multiple SNPs jointly modify the phenotype. These maximal connected subgraphs show various structural patterns, might implying the existence of unique interaction patterns among groups of SNPs. In the hypergraph, the largest one consists of 31 SNPs displaying a tree-like structure. Almost all these 31 SNPs have small main effects, but their total effects may be reinforced through hub SNPs and other connectivity structures in the hypergraph. Hub SNPs, for example, rs10518739, may be important to the phenotype, not because of their individual effects, but because of overall influence in modulating the effects of other SNPs. Further analyses of these maximal connected subgraphs are necessary, although they are beyond the scope of this study. We hope that, from these experiments, some clues could be provided for the exploration of causative factors of AMD.

## Conclusions

Epistatic interactions are believed to play an increasingly important role in unraveling the mystery of “missing heritability”, and detection of them has already become a compelling step in GWAS. Though many works have been done for their detection, most only focus on pairwise epistatic interactions due to the methodological and computational challenges. In this study, we introduce a methodology CINOEDV for the detection of multiple epistatic interactions with different orders. CINOEDV is a two stage method: detecting stage for identifying SNPs with high main effects and *n*-order epistatic interactions with high *n*-order interaction effects, visualizing stage for visualizing the detected epistatic interactions and capturing higher order epistatic interactions. In detecting stage, two co-information based measures, namely, *NCI* and *CCI*, are developed for quantifying effects of *n*-order SNP combinations to the phenotype, and two types of search strategies are provided for dealing with different situations: the exhaustive search for lower order epistatic interactions and/or small scale data sets, the PSO based search for higher order epistatic interactions and/or large scale data sets. In visualizing stage, all detected SNPs and their corresponding *n*-order interaction effects are used to construct an epistasis hypergraph, where a real vertex denotes the main effect of a SNP and a virtual vertex represents the *n*-order interaction effect of its linking SNPs. This hypergraph is able to supervise the search for higher order epistatic interactions at a substantially reduced computational cost. Experiments of CINOEDV and its comparison with state-of-the-art methods, including TEAM, BOOST, SNPRuler, AntEpiSeeker, *epi*MODE, GAIN, SEN, ViSEN, and EINVis, are performed on lots of simulation data sets. Results demonstrate that CINOEDV is promising in detecting and visualizing multiple epistatic interactions with different orders. CINOEDV is also applied on a real AMD data set, results of which not only show the strength of CINOEDV on real applications, but also capture important features of genetic architecture of AMD that have not been described previously. These features might provide new clues for biologists on the exploration of AMD-associated genetic factors.

CINOEDV is implemented in R and is freely available from R CRAN (http://cran.r-project.org) and the website (https://sourceforge.net/projects/cinoedv/files/). It is a user-friendly cross-platform software package. Its input data are stored in a MAT format to accommodate large data sets, and a few parameters should be set according to their recommendation options. The generated effect lists, or similar lists produced by other software, are exported for epistasis hypergraph construction, which implies that components of CINOEDV package can be used independently, facilitating wide adoption of CINOEDV. Furthermore, several useful tools are also provided for deep analyses of the constructed hypergraph. More details of CINOEDV package are in user manual.

CINOEDV might be an alternative to existing methods for the detection and visualization of *n*-order epistatic interactions, and has several advantages.

First, different from existing methods, such as BOOST, AntEpiSeeker, SNPRuler, *epi*MODE, and TEAM, mainly focusing on the detection of pairwise epistatic interactions, and easily ignoring the broader epistasis landscape, CINOEDV is able to discover multiple epistatic interactions with their orders from 2 to *n* simultaneously, where *n* is the specified maximum order and can be set to 3 or larger.

Second, CINOEDV creates a global interaction map that not only shows all orders of effects at the same time, where effects can be compared intuitively and fairly, but also distinguish interaction effects of different orders from main effects effectively, which is important to find the dominant effect in modifying the phenotype.

Third, the epistasis hypergraph constructed by main effects and low order interaction effects is a promising guide map for capturing higher order epistatic interactions while substantially reducing computational cost, which implies that CINOEDV can handle large scale data sets. This reduction of computational complexity is even more encouraging in the era of GWAS.

Fourth, CINOEDV is capable of visualizing detected epistatic interactions and their interaction effects of different orders in the hypergraph. As far as we know, it is the first visualization software that shows *n* (i.e., *n* = 5) orders of effects simultaneously. Such an idea embracing the complexity of genetic architecture underlying complex diseases, may contribute to better understand the detected epistatic interactions, capture global epistasis landscape, depict their unique interacting patterns.

Fifth, the hypergraph may be useful for revealing more clues to interpret the mechanism of a complex disease, for example, hub SNPs, connected subgraphs, density subgraphs, and many others. These graph structures cannot be captured by traditional methods.

Though CINOEDV is a beneficial exploration in detecting and visualizing epistatic interactions, it still has several limitations, and needs further improvement, innovation and development, which inspire us to continue working in the future.

First, the exhaustive search is heavy in time cost, and the PSO based search loses the calculation accuracy though it can finish the work at affordable time cost. How to balance the calculation accuracy and the time cost is a direction.

Second, hypergraph cannot supervise to infer a pure high order epistatic interaction displaying no lower order interaction effects and no marginal effects.

Third, hypergraph analyses are sample in the paper. However, it is believed that more detailed analyses of the hypergraph, for example, pathway enrichment analysis, integrative analysis and others, might capture more important clues.

Last but not the least, current co-information based association measures in CINOEDV only consider binary discrete traits. It is important to extend the association measures to continuous traits.

### Ethics approval and consent to participate

Not applicable.

### Consent for publication

Not applicable.

### Availability of data and material

The simulation data sets supporting the conclusions of this article are available online at http://www.bdmb-web.cn/index.php?m=content&c=index&a=lists&catid=28. The AMD data set is available from the original article, which is cited in this article.

## Additional file

Additional file 1: The note describes evaluation measures, such as detection power and computational complexity. Additional file 1: Figure S1 – S4 record GAIN, SEN, ViSEN, and EINVis results of compared simulation data sets, respectively. Additional file 1: Table S1 - S3 report top 20 SNPs with high main effects, top 20 epistatic interactions with high 2-order interaction effects, and top 20 SNPs with high 3-order interaction effects on AMD data set. Additional file 1: Table S4 lists the detected SNPs and epistatic interactions of other competing methods on AMD data set. (PDF 850 kb)

## Abbreviations

SNPs: single nucleotide polymorphisms
GWAS: genome-wide association studies
GAIN: genetic association interaction network
SEN: statistical epistasis network
PSO: particle swarm optimization
AMD: age-related macular degeneration
