# Supplementary material for: CINOEDV: a co-information based method for detecting and visualizing n-order epistatic interactions
Source: BMC Bioinformatics. 2016 May 17;17:214. doi: 10.1186/s12859-016-1076-8 (PMC4869388; doi:10.1186/s12859-016-1076-8)
Supplement: Additional file 1: — The note describes evaluation measures, such as detection power and computational complexity. Additional file 1: Figure S1 – S4 record GAIN, SEN, ViSEN, and EINVis results of compared simulation data sets, respectively. Additional file 1: Table S1 - S3 report top 20 SNPs with high main effects, top 20 epistatic interactions with high 2-order interaction effects, and top 20 SNPs with high 3-order interaction effects on AMD data set. Additional file 1: Table S4 lists the detected SNPs and epistatic interactions of other competing methods on AMD data set. (PDF 850 kb) [file 12859_2016_1076_MOESM1_ESM.pdf]

CINOEDV: a co-information based method for detecting and visualizing *n*-order epistatic interactions – Supplementary file

Junliang Shang[1,2,*],Yingxia Sun[1], Jin-Xing Liu[1], Junfeng Xia[3], Junying Zhang[4], Chun-Hou Zheng[5]

[1]School of Information Science and Engineering, Qufu Normal University, Rizhao 276826, China, [2]Institute of Network Computing, Qufu Normal University, Rizhao 276826, China, [3]Institute of Health Sciences, Anhui University, Hefei, Anhui 230601, China, [4]School of Computer Science and Technology, Xidian University, Xi'an 710071, China, [5]College of Electrical Engineering and Automation, Anhui University, Hefei, Anhui 230039, China

*To whom correspondence should be addressed.

NOTE 1. EVALUATION MEASURES (DETECTION POWER AND COMPUTATIONAL COMPLEXITY).

In the study, detection power and computational complexity are applied to evaluate the performance of CINOEDV.

Various forms of detection power have been proposed depending on what is desired to measure [1-9], and three types of them are introduced with constraints from conservative to modest.

Power1 is defined as the proportion of data sets in which all ground-truth SNPs are identified with no false positives where ground-truth SNPs refer to the causative SNPs that truly associated with the phenotype, in other words, the SNPs in models added into the simulation data sets. Power1 can be written as

$$\text{Power1} = \frac{1}{D}\sum_{i=1}^{D} x_i ,$$

where D is the number of simulation data sets with the same parameter settings, and $x_i \in \{0,1\}$ is the detection tag, i.e., if all ground-truth SNPs in data set i are detected with no false positives, $x_i = 1$, otherwise, $x_i = 0$. Though Power1 seems not practical since false positives are inevitable for any statistical tests and fewer false positives result in larger false negatives, we still introduce it because it is advantageous in practical applications, and might interest biologists due to false positives implying wasted experimental effort to validate the results.

Sometimes, allowing some small type-I error is more reasonable, thus Power2 and Power3 are introduced. Power2 and Power3 are defined as an average proportion of ground-truth SNPs in the top L_i detected SNPs, and can be respectively written as

$$\text{Power2} = \frac{1}{D}\sum_{i=1}^{D} \frac{y_i}{L_i} ,$$

$$\text{Power3} = \frac{1}{D}\sum_{i=1}^{D} \frac{z_i}{L_i} ,$$

where y_i and z_i are the numbers of ground-truth SNPs appearing in the top L_i SNPs identified in data set i. For Power2, L_i is equal to the number of ground-truth SNPs in data set i. For Power3, L_i is normally set to a value larger than that in Power2. In our experiments, L_i is set to 4.

Computational complexity is also considered. Running time in the same computational environment is measured to assess realistic applicability of compared methods.

Figure S1. GAIN RESULTS OF COMPARED DATA SETS.

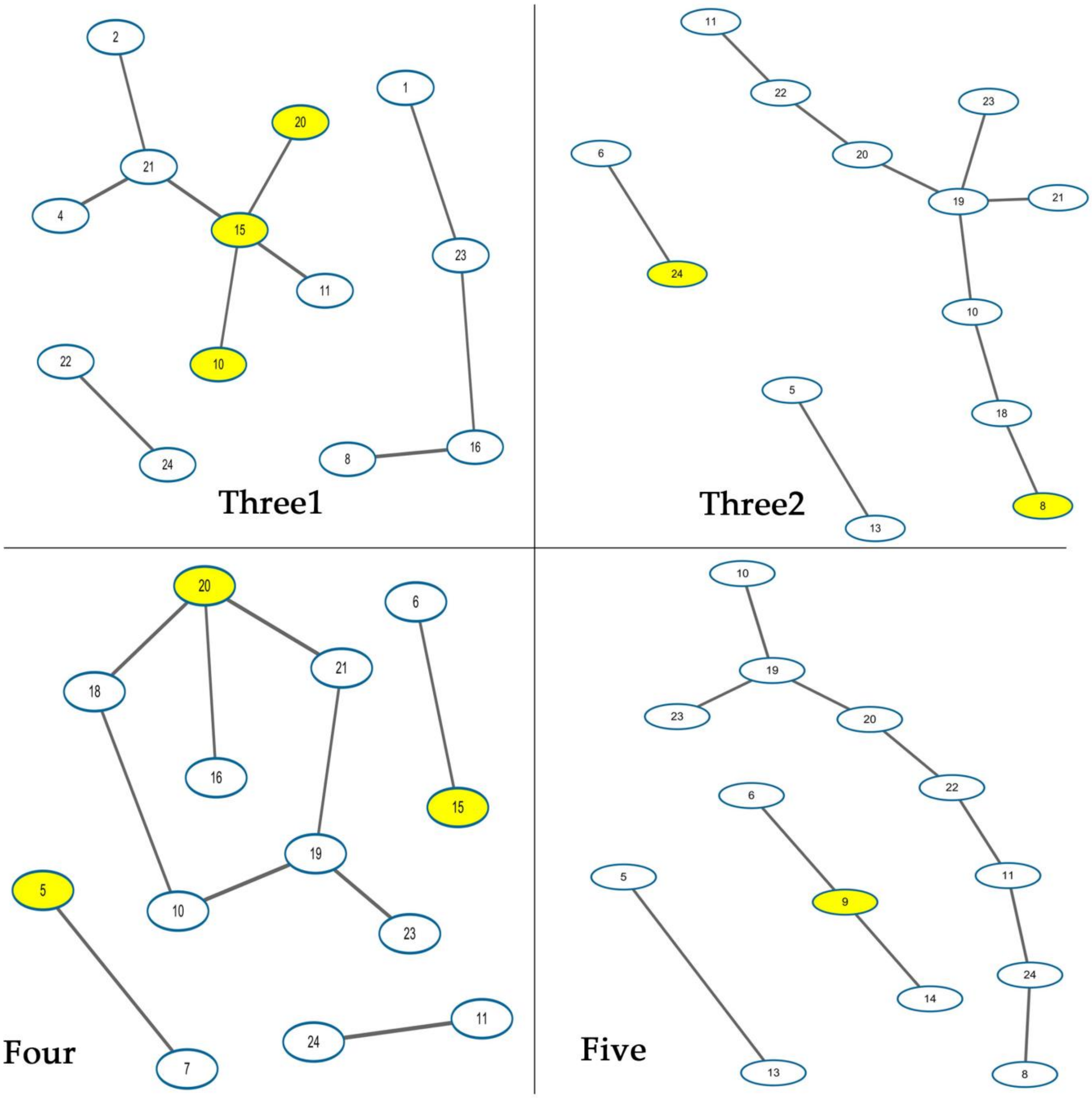

Figure S2. SEN RESULTS OF COMPARED DATA SETS.

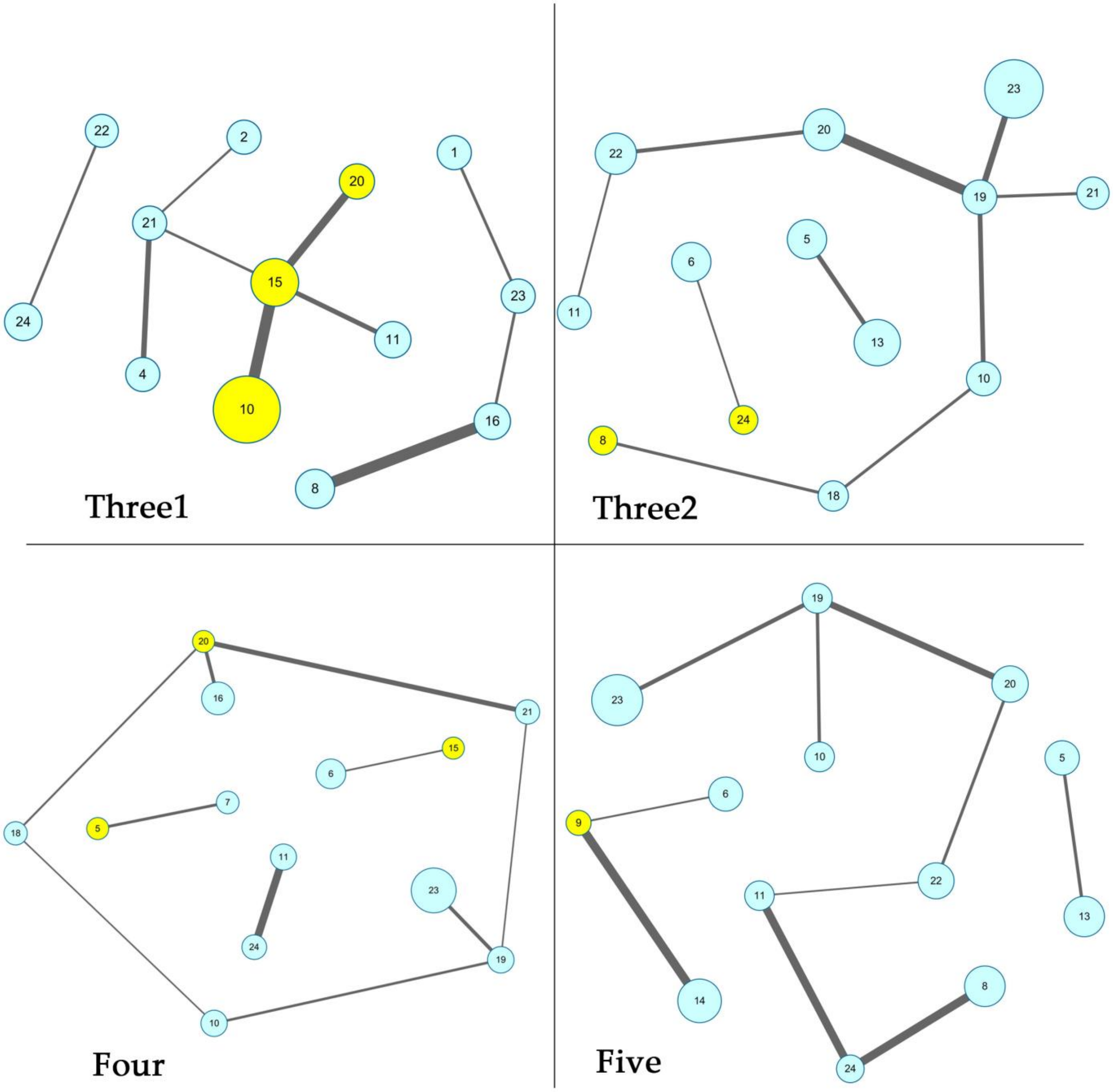

Figure S3. VISEN RESULTS OF COMPARED DATA SETS.

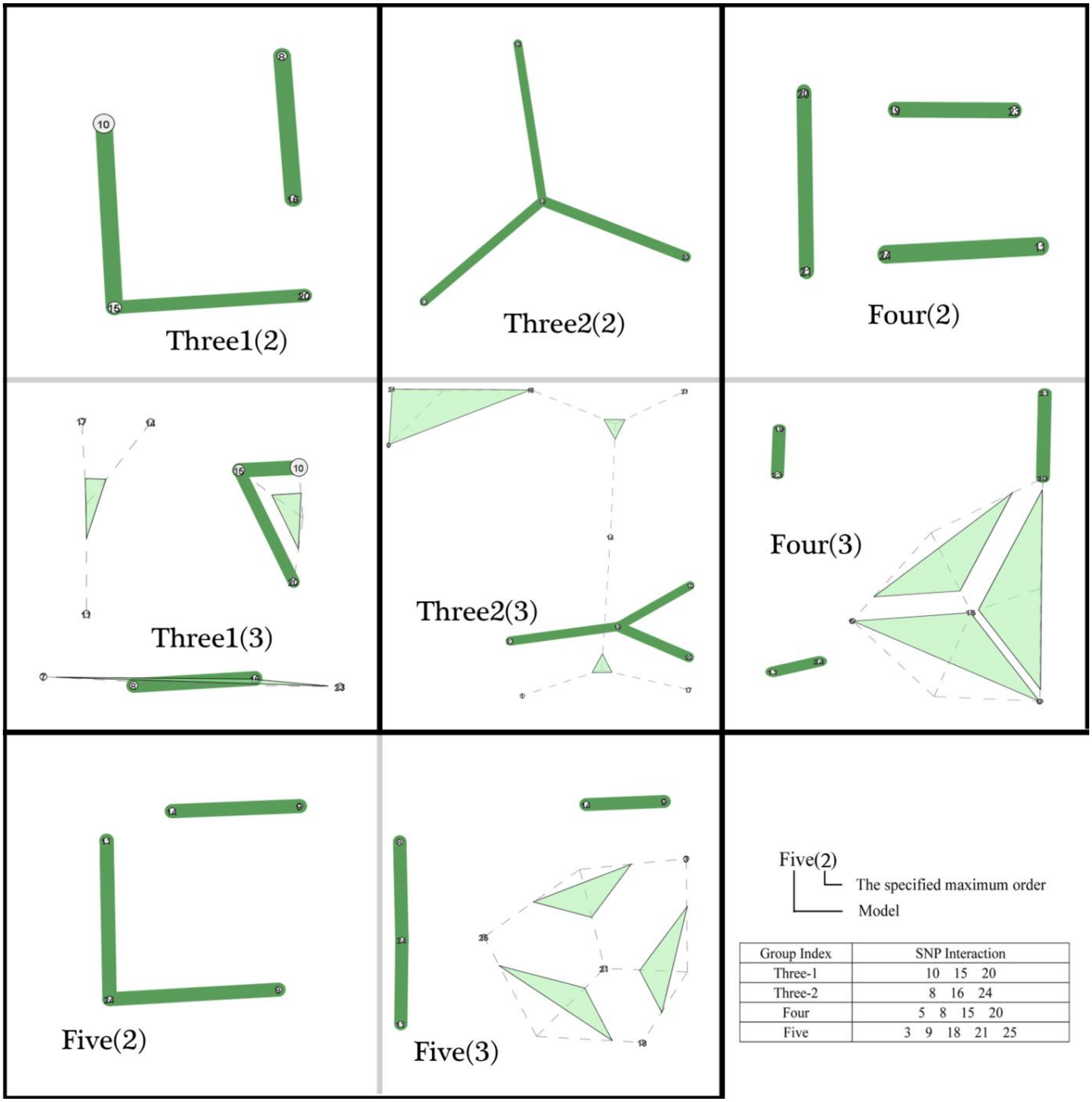

Group Index	SNP Interaction
Three-1	10 15 20
Three-2	8 16 24
Four	5 8 15 20
Five	3 9 18 21 25

Figure S4. EINVIS RESULTS OF COMPARED DATA SETS.

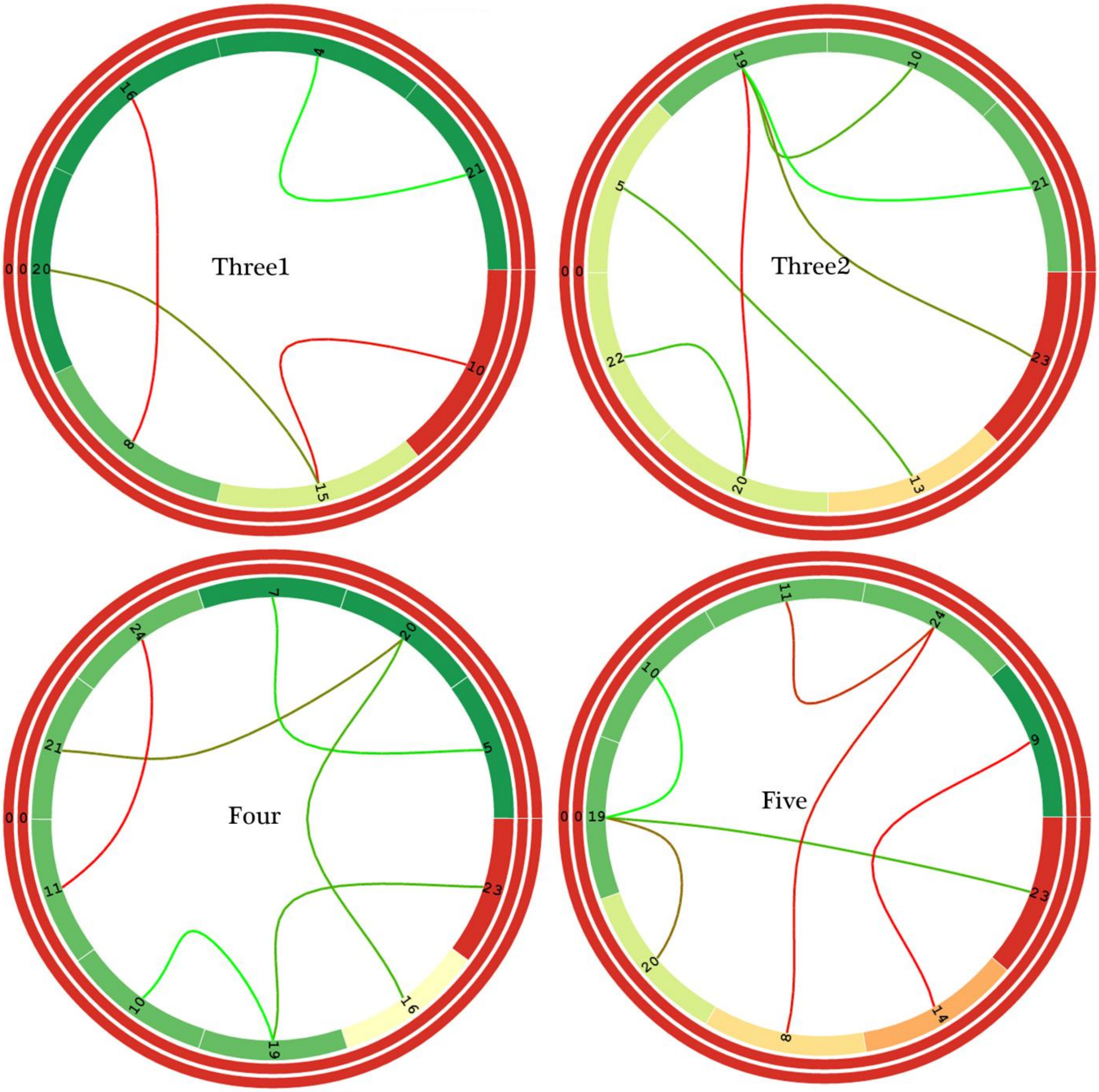

Table S1. **TOP 20 SNPS WITH HIGH MAIN EFFECTS.**

For the columns “Index” in Table S2, Table S3 and Table S4, bold and underlined numbers represent that their corresponding epistatic interactions are the top 20 ones with the high *CCI* values. For the columns “SNP” in Table S2, and “Epistatic Interactions” in Table S3 and Table S4, bold SNPs are those in the largest maximal connected subgraph of epistasis hypergraph Figure S1 of main paper, and underlined SNPs are those in the second largest maximal connected subgraph of epistasis hypergraph Figure S1 of main paper.

Index	SNP	Gene	Chromosome	*NCI* Value
1	rs380390	*CFH*	Chr1	0.06472
2	rs1329428	*CFH*	Chr1	0.05857
3	rs3775652	*INPP4B*	Chr4	0.05696
4	rs1930022	*N/A*	Chr9	0.04858
5	**rs1394608**	*SGCD*	Chr5	0.04562
6	rs725518	*RRM1*	Chr11	0.04496
7	rs10511467	*N/A*	Chr9	0.04485
8	rs1740752	*N/A*	Chr10	0.04434
9	rs10507949	*N/A*	Chr13	0.04345
10	<u>rs9294603</u>	*N/A*	Chr6	0.04184
11	rs6967345	*N/A*	Chr7	0.04181
12	rs3913094	*N/A*	Chr12	0.04076
13	**rs7006908**	*N/A*	Chr8	0.04025
14	rs10254116	*BBS9*	Chr7	0.04019
15	rs3914244	*N/A*	Chr12	0.04018
16	rs7104698	*N/A*	Chr11	0.03961
17	rs10501439	*TENM4*	Chr11	0.03958
18	rs7863587	*N/A*	Chr9	0.03882
19	rs10512174	*ISCA1*	Chr9	0.03870
20	rs1363688	*N/A*	Chr5	0.03867

Table S2. **TOP 20 EPISTATIC INTERACTIONS WITH HIGH 2-ORDER INTERACTION EFFECTS.**

For the columns “Index” in Table S2, Table S3 and Table S4, bold and underlined numbers represent that their corresponding epistatic interactions are the top 20 ones with the high *CCI* values. For the columns “SNP” in Table S2, and “Epistatic Interactions” in Table S3 and Table S4, bold SNPs are those in the largest maximal connected subgraph of epistasis hypergraph Figure S1 of main paper, and underlined SNPs are those in the second largest maximal connected subgraph of epistasis hypergraph Figure S1 of main paper.

Index	Epistatic Interaction	Gene	Chromosome	*NCI* Value
**1**	**rs994542**	*N/A*	Chr6	0.25762
	**rs9298846**	*N/A*	Chr9	
**2**	rs2359907	*N/A*	Chr1	0.22561
	rs9326913	*N/A*	Chr5	
**3**	rs2957935	*SKAP1*	Chr17	0.22267
	rs10519745	*N/A*	Chr5	
**4**	rs10231824	*PLXNA4*	Chr7	0.20243
	rs571361	*N/A*	Chr1	
**5**	rs4856145	*N/A*	Chr3	0.19749
	rs3828057	*RORC*	Chr1	
**6**	rs1436532	*N/A*	Chr4	0.19140
	rs10507601	*N/A*	Chr13	
**7**	rs305728	*N/A*	Chr5	0.19057
	rs10497071	*N/A*	Chr2	
**8**	**rs2298809**	*N/A*	Chr11	0.18960
	**rs7688514**	*VEGFC*	Chr4	
**9**	**rs2278031**	*AP1G1*	Chr16	0.18154
	**rs446815**	*N/A*	Chr11	
**10**	rs756308	*N/A*	Chr10	0.18122
	rs2011542	*N/A*	Chr7	
**11**	rs10516495	*SLC9B1*	Chr4	0.18122
	rs2011542	*N/A*	Chr7	
**12**	rs339884	*N/A*	Chr18	0.18119
	rs195937	*N/A*	Chr4	
**13**	**rs1408120**	*PTPRD*	Chr9	0.17685
	**rs10506115**	*N/A*	Chr12	
**14**	rs595113	*N/A*	Chr1	0.16972
	rs9300104	*RNF141*	Chr11	
**15**	**rs10518739**	*MAP2K5*	Chr15	0.16603
	**rs931210**	*N/A*	Chr5	
**16**	**rs2386906**	*N/A*	Chr10	0.16543
	**rs9313446**	*SLIT3*	Chr5	
**17**	**rs9313446**	*SLIT3*	Chr5	0.16349
	**rs293379**	*ABHD2*	Chr15	
18	rs1458402	*N/A*	Chr6	0.15851
	rs4901408	*N/A*	Chr14	
19	rs6483628	*NAV2*	Chr11	0.15589
	rs10504003	*N/A*	Chr8	
20	**rs3844556**	*PKD2L1*	Chr10	0.15405
	**rs10521129**	*ZZEF1*	Chr17	

Table S3. **TOP 20 EPISTATIC INTERACTIONS WITH HIGH 3-ORDER INTERACTION EFFECTS.**

For the columns "Index" in Table S2, Table S3 and Table S4, bold and underlined numbers represent that their corresponding epistatic interactions are the top 20 ones with the high *CCI* values. For the columns "SNP" in Table S2, and "Epistatic Interactions" in Table S3 and Table S4, bold SNPs are those in the largest maximal connected subgraph of epistasis hypergraph Figure S1 of main paper, and underlined SNPs are those in the second largest maximal connected subgraph of epistasis hypergraph Figure S1 of main paper.

Index	Epistatic Interaction	Gene	Chromosome	*NCI* Value
**<u>1</u>**	**rs4723261**	*PTHB1*	Chr7	0.17507
	**rs1036995**	*PCDH9*	Chr13	
	**rs2298809**	*N/A*	Chr11	
**<u>2</u>**	<u>rs595113</u>	*N/A*	Chr1	0.16096
	<u>rs2031175</u>	*ANKRD15*	Chr9	
	<u>rs9300104</u>	*RNF141*	Chr11	
**<u>3</u>**	rs10517546	*N/A*	Chr4	0.15934
	rs10500875	*N/A*	Chr11	
	rs10505107	*ANGPT1*	Chr8	
4	**rs2386906**	*N/A*	Chr10	0.14875
	**rs1476623**	*NXPH1*	Chr7	
	**rs10518739**	*MAP2K5*	Chr15	
5	**rs293379**	*ABHD2*	Chr15	0.14681
	**rs1476623**	*NXPH1*	Chr7	
	**rs10518739**	*MAP2K5*	Chr15	
6	<u>rs1436532</u>	*N/A*	Chr4	0.14445
	<u>rs9294603</u>	*N/A*	Chr6	
	<u>rs595113</u>	*N/A*	Chr1	
7	**rs1394608**	*SGCD*	Chr5	0.14439
	**rs9323782**	*N/A*	Chr14	
	**rs931210**	*N/A*	Chr5	
8	<u>rs1374431</u>	*N/A*	Chr2	0.14414
	<u>rs1436532</u>	*N/A*	Chr4	
	<u>rs1978419</u>	*N/A*	Chr5	
9	rs3922799	*N/A*	Chr2	0.13967
	rs10517546	*N/A*	Chr4	
	rs10500875	*N/A*	Chr11	
10	**rs994542**	*N/A*	Chr6	0.13863
	**rs3781868**	*NPAT*	Chr11	
	**rs2207768**	*NRG3*	Chr10	
11	**rs1394608**	*SGCD*	Chr5	0.13813
	**rs1408120**	*PTPRD*	Chr9	
	**rs10495668**	*SMC6*	Chr2	
12	**rs1926489**	*GPC5*	Chr13	0.13730
	**rs1408120**	*PTPRD*	Chr9	
	**rs718309**	*PCDH15*	Chr10	
13	<u>rs10486519</u>	*PTHB1*	Chr7	0.13669
	<u>rs1978419</u>	*N/A*	Chr5	
	<u>rs10507601</u>	*N/A*	Chr13	
14	**rs7006908**	*N/A*	Chr8	0.13636
	**rs10518739**	*MAP2K5*	Chr15	
	**rs10495668**	*SMC6*	Chr2	
15	**rs293379**	*ABHD2*	Chr15	0.13526
	**rs9287251**	*RGS7*	Chr1	
	**rs446815**	*N/A*	Chr11	
16	**rs2182277**	*N/A*	Chr6	0.13441
	**rs1036995**	*PCDH9*	Chr13	

	**rs2207768**	*NRG3*	Chr10	
17	**rs3844556**	*PKD2L1*	Chr10	0.13419
	**rs1036995**	*PCDH9*	Chr13	
	**rs4880042**	*PAX5*	Chr9	
18	**rs2386906**	*N/A*	Chr10	0.13324
	**rs9287251**	*RGS7*	Chr1	
	**rs446815**	*N/A*	Chr11	
19	**rs3743175**	*ZNF291*	Chr15	0.13317
	**rs9323782**	*N/A*	Chr14	
	**rs4880042**	*PAX5*	Chr9	
20	**rs2828155**	*N/A*	Chr21	0.13317
	**rs9323782**	*N/A*	Chr14	
	**rs4880042**	*PAX5*	Chr9	

Table S4. DETECTED SNPS AND EPISTATIC INTERACTIONS OF OTHER COMPETING METHODS.

In the table, p-values are calculated by the *chi*-squared test. *N/A* means no gene being available. Detected SNPs and epistatic interactions of both BEAM and *epi*MODE are directly cited from their respective papers.

Method	SNP (Epistatic Interaction)	P-value	Gene	Chromosome
SNPRuler	rs10499664	1.099e-05	*N/A*	Chr7
	rs10510608		*MGC61571*	Chr3
	rs10493240	3.117e-05	*N/A*	Chr1
	rs10512484		*N/A*	Chr17
	rs10485289	3.535e-05	*N/A*	Chr6
	rs10513713		*N/A*	Chr3
	rs727113	4.038e-05	*N/A*	Chr13
	rs9284239		*N/A*	Chr13
	rs1331977	4.461e-05	*APBA1*	Chr9
	rs10506115		*N/A*	Chr12
	rs2135488	4.468e-05	*N/A*	Chr13
	rs6772323		*KCNAB1*	Chr3
	rs727922	9.632e-05	*CAMK4*	Chr5
	rs2549513		*N/A*	Chr16
	rs4674305	1.013e-04	*VIL1*	Chr2
	rs10513027		*N/A*	Chr5
	rs3798425	1.750e-04	*MY06*	Chr6
	rs692897		*COP1*	Chr11
	rs10487321	2.066e-04	*N/A*	Chr7
	rs1331977		*APBA1*	Chr9
AntEpiSeeker	**rs380390**	1.752e-06	*CFH*	Chr1
	rs10511901	6.829e-04	*N/A*	Chr9
	rs10488343	1.100e-03	*N/A*	Chr7
	s10505093	2.800e-03	*N/A*	Chr8
	rs3844556	1.473e-06	*PKD2L1*	Chr10
	rs10507949		*N/A*	Chr13
	rs10492225	4.250e-05	*N/A*	Chr12
	rs176880		*ZNF37A*	Chr10
	rs724820	5.901e-05	*C20orf133*	Chr20
	rs2143881		*N/A*	Chr6
	**rs380390**	6.500e-05	*CFH*	Chr1
	rs944838		*GPC6*	Chr13
	rs10484309	9.678e-05	*N/A*	Chr6
	rs10254116		*PTHB1*	Chr7
BEAM	**rs380390**	1.752e-06	*CFH*	Chr1
	**rs1329428**	3.643e-06	*CFH*	Chr1
*epi*MODE	rs3743175	7.398e-07	*ZNF291*	Chr15
	**rs1394608**		*SGCD*	Chr1
	rs3743175	1.341e-05	*ZNF291*	Chr15
	rs2828155		*N/A*	Chr21
	rs10507949		*N/A*	Chr13
	**rs1394608**		*SGCD*	Chr1
	rs3886593	3.698e-05	*MSI2*	Chr17
	rs10491243		*N/A*	Chr5
	**rs1329428**		*CFH*	Chr1
	**rs380390**		*CFH*	Chr1
BOOST	**rs380390**	1.752e-06	*CFH*	Chr1
	**rs1329428**	3.643e-06	*CFH*	Chr1
	rs3775652	4.152e-05	*INPP4B*	Chr4
	**rs1394608**	8.806e-05	*SGCD*	Chr1

rs1930022	2.194e-04	*N/A*	Chr9
rs994542	**5.068e-09**	*N/A*	Chr6
rs9298846		*N/A*	Chr9
rs2359907	1.242e-06	*N/A*	Chr1
rs9326913		*N/A*	Chr5
rs2957935	1.627e-06	*SKAP1*	Chr17
rs10519745		*N/A*	Chr5
rs2386906	1.915e-05	*N/A*	Chr10
rs9313446		*SLIT3*	Chr5
rs293379	2.017e-05	*ABHD2*	Chr15
rs9313446		*SLIT3*	Chr5

REFERENCES

1. Zhang Y, Liu JS: **Bayesian inference of epistatic interactions in case-control studies**. *Nature Genetics* 2007, **39**(9):1167-1173.
2. Shang J, Zhang J, Sun Y, Zhang Y: **EpiMiner: A three-stage co-information based method for detecting and visualizing epistatic interactions**. *Digital Signal Processing* 2014, **24**:1-13.
3. Wan X, Yang C, Yang Q, Xue H, Fan X, Tang NLS, Yu W: **BOOST: A fast approach to detecting gene-gene interactions in genome-wide case-control studies**. *American Journal of Human Genetics* 2010, **87**(3):325.
4. Wan X, Yang C, Yang Q, Xue H, Tang NL, Yu W: **Predictive rule inference for epistatic interaction detection in genome-wide association studies**. *Bioinformatics* 2010, **26**(1):30-37.
5. Wang Y, Liu X, Robbins K, Rekaya R: **AntEpiSeeker: detecting epistatic interactions for case-control studies using a two-stage ant colony optimization algorithm**. *BMC Research Notes* 2010, **3**(1):117.
6. Tang W, Wu X, Jiang R, Li Y: **Epistatic module detection for case-control studies: a Bayesian model with a Gibbs sampling strategy**. *PLoS genetics* 2009, **5**(5):e1000464.
7. Shang J, Zhang J, Sun Y, Liu D, Ye D, Yin Y: **Performance analysis of novel methods for detecting epistasis**. *BMC Bioinformatics* 2011, **12**(1):475.
8. Shang J, Zhang J, Lei X, Zhang Y, Chen B: **Incorporating heuristic information into ant colony optimization for epistasis detection**. *Genes & Genomics* 2012, **34**(3):321-327.
9. Aflakparast M, Salimi H, Gerami A, Dubé M, Visweswaran S, Masoudi-Nejad A: **Cuckoo search epistasis: a new method for exploring significant genetic interactions**. *Heredity* 2014, **112**(6):666-674.
